# From Stories to Solutions: A Research Cycle Framework for Enhancing Trustworthiness in Studies of Online Patient Narratives

**DOI:** 10.2196/58310

**Published:** 2025-01-23

**Authors:** Klay Lamprell, Diana Fajardo Pulido, Gaston Arnolda, Bróna Nic Giolla Easpaig, Yvonne Tran, Jeffrey Braithwaite

**Affiliations:** 1 Macquarie University Australian Institute of Health Innovations Sydney Australia; 2 Portal Pontificia Universidad Javeriana Bogotá Colombia; 3 School of Nursing Charles Darwin University Darwin Australia; 4 Macquarie University Hearing Macquarie University Sydney Australia

**Keywords:** online research, exploratory study, patient experience, patient narratives, narrative analysis, mixed methods, young-onset colorectal cancer, cancer, oncology, internal medicine

## Abstract

Personal accounts of health care experiences posted to online platforms are a rich source of patient-reported data. Noninteractive narratives, published as one-off accounts, often describe an entire patient journey in one story, featuring transitions through health care settings from prediagnosis to outcome. These coherent perspectives may not be visible or captured in fragmented social media posts or episodic blogs. For researchers seeking patient-reported accounts of care quality across a clinical trajectory, noninteractive online narratives can be an invaluable, easily accessible resource. The challenges and limitations of research utilizing these data may differ from working with interactive patient narratives and include source transparency and credibility, limited or no information about authors, and ambiguity about the health care context and time frames. We outline a framework for addressing these issues in 5 key phases of the research cycle: the conceptual-theoretical approach; locating sources of data; accessing and collecting the data; quantitative profiling to establish demographics, health care events, and time frames; and qualitative analysis utilizing multiple modes of narrative inquiry. We explain the use of this framework in a real-world example: our study of 273 online patient narratives describing health care trajectories in early-onset bowel cancer.

**International Registered Report Identifier (IRRID):**

RR2-10.2196/25056

## Introduction

Online patient narratives have been categorized as interactive or noninteractive [1,2], each offering distinct advantages and challenges for health care research. Interactive narratives, typically shared through social media, blogs, and forums, allow patients to update their stories, respond to feedback, and report evolving experiences. Noninteractive or static narratives, published to websites as one-off reports, typically encapsulate the entire patient journey over time. This coherent, patient-framed perspective on the progression from prediagnosis to outcome may not be visible or captured in the fragmented or episodic formats of interactive narratives.

For researchers investigating patient-framed perceptions of care across a clinical trajectory, noninteractive online narratives, which can also be referred to as online pathographies [3,4], offer an invaluable resource. The holistic narrative structure allows the representation of both a psychological transition and a physical movement through various phases of biomedical intervention and health care settings. They can expose health service delays and continuity gaps that only patients can observe as sole witnesses to the entirety of their experiences. Importantly, the global availability of online patient narratives allows researchers ease of access and, potentially, insights into the diversity of health system experiences.

The value of these pathographies for researchers must be weighed against unique challenges to validity and trustworthiness that share common elements with, but also extend beyond, typical issues in qualitative research, and potentially even beyond challenges with the use of interactive narratives. Noninteractive online narratives present risks such as source credibility, limited or no information about authors, and uncertainty about the health care context and time frames. These aspects of the data may present constraints on analytic interpretation.

We navigated these challenges in an exploratory study to understand the causes of delays in the diagnosis of early-onset bowel cancer, defined as a diagnosis in patients younger than the age of 50 years [5]. Our study drew on narratives published on prominent bowel cancer support websites in the United Kingdom, Australia, and New Zealand, published under banners such as “Real life stories” and “Your Story” [6,7]. The findings of our study, reported elsewhere [6,7], highlighted a critical need to lower the threshold age for public bowel cancer screening programs and revise clinical guidelines for diagnostic pathways.

**Figure 1:**
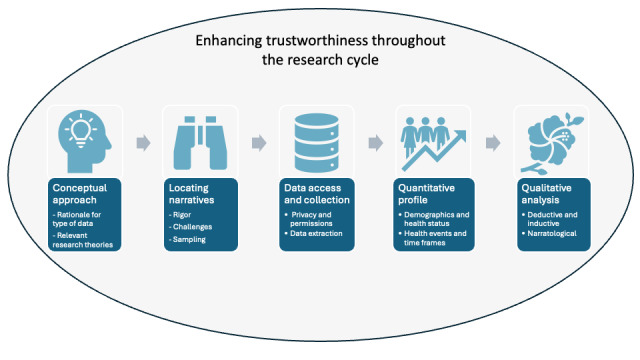
A framework of five key focal points in the research cycle for addressing the credibility and enhancing the trustworthiness of research involving noninteractive, online patient narratives.

To help structure the research process in ways that enhance reliability, we developed a framework featuring 5 key phases throughout the research cycle requiring heightened attention to issues of validity and reliability (see Figure 1). The framework developed by Zoolnoori et al [8], for use with interactive online patient narratives, focuses on 4 of the main phases of research: data collection, data preparation, content analysis, and interpretation of the results. We found that a different set of focal points were needed to address the unique challenges of research investigating noninteractive online narratives. Below, we describe each of the 5 focal points in our framework through the lens of our work with noninteractive, early-onset bowel cancer narratives.

### Ethical Considerations

Ethical and scientific approval was granted by Macquarie University Human Research Ethics Committee (reference 52020666115757).

### Conceptual-Theoretical Approach

The framework begins in the conceptualizing phase, establishing a rationale for the use of online pathographies and highlighting a detailed theoretical framework for the research. **As** exploratory research, the rationale for utilizing online patient pathographies was to determine the relevance of progressing to purposively sampled studies [9]. The rationale was further supported by literature indicating that patients younger than the age of 50 years are drawn to, and are adept at using, the internet for information seeking and sharing [10].

Narrative inquiry is an apt overarching methodology for research that leverages online narratives; it focuses on exploring and interpreting phenomena through stories of lived experience [11] and features flexibility in blending qualitative and quantitative elements to support trustworthy findings [12,13]. Key theories validating singular narratives as evidence of phenomena [14] include constructivism (knowledge through experiences), phenomenology (shared experiences), hermeneutics (text interpretation), and narratology (narrative structures).

### Locating Authentic Narratives

We included a focal point on the source of the pathographies because the **credibility** and **authenticity** of noninteractive narratives are closely tied to the reliability of the source. It is crucial to establish some means of locating pathographies that can be assumed to be authentic. We looked to institutional oversight as a way to verify authenticity. We followed the Internet Mediated Research (IMR) search strategy outlined by Obrien and Clark [15], using comprehensive search terms and multiple platforms, as well as systematically applying inclusion and exclusion criteria to locate relevant pathographies published on the websites of well-established institutions. We excluded pathographies from health care organization websites that presented exclusively positive views for marketing purposes.

Demographics and clinical information about the authors were typically embedded within the narratives and not necessarily explicitly stated; we had to review all pathographies identified on validated sites line by line to confirm they met the criteria for early-onset colorectal cancer.

### Data Access and Collection

We highlight this phase in the research cycle because the unsolicited, often anonymous, and noninteractive nature of the data necessitated careful negotiation of consent. We contacted each organization to ensure that the conditions for consent were met and to obtain explicit approval for empirical investigation and dissemination of our findings. Permission from individual authors was not required, aligning with the principles of “unobtrusive” IMR [16,17] and the concept of “human-generated data” [18], which individuals willingly produce as they engage in various online and offline activities. This approach met the ethical guidelines of the Australian National Statement on Ethical Conduct in Human Research 2007 (Updated 2018) [19] and the British Psychological Society’s principles for IMR [20].

Where technically possible, we used the website’s export options to extract narrative text from the online platform and download it into an analysis software program, NVivo 12 (version 12; QSR International Pty Ltd). Alternatively, we used a cut-and-paste function, creating a Microsoft Word document for each narrative, which was then imported into NVivo.

### Quantitative Profiling

We specify that a quantitative approach is needed to resolve the likely anonymity of authors. As noted, authors’ demographics and health status characteristics were not always evident in the unsolicited data, which presented the risk of over- and underrepresentation of subpopulations and particular types of diagnostic experiences. To ensure the transparency of any data bias, we used NVivo to quantify the demographic and health status information in the narratives and cataloged the *type*, *frequency*, and *time frames* of diagnostic events described in the texts, including first event of seeking help for symptoms, types of tests and screening events, and referrals to specialists.

In extracting demographic and health status data from narratives and cataloging diagnostic events by type, frequency, and time frame, we identified a gender disparity of authorship, with over 70% of narratives written by people identifying as female, despite approximately equal gender diagnoses of early-onset colorectal cancer [21]. Additionally, the quantitative profile identified that most narratives were written by people aged younger than 40 years, although there is a higher incidence of early-onset colorectal cancer in the 40-49 years age group. This finding highlights the relevance of online methods when investigating younger populations but acknowledges the potential for bias when generalizing across sets of unsolicited data. In our reporting of our findings, we openly discussed these biases and hypothesized others, such as potential underrepresentation of culturally and linguistically diverse and special needs populations.

### Qualitative Analysis

We assert that manual qualitative analysis of pathographies continues to offer the greatest potential for sensitivity to textual nuances and tonal idiosyncrasies indicating facilitators and barriers to quality care over time [8]. Pathographies embed time in 3 key narrative layers: the subjective account of chronological time, detailing the sequence of events from symptom onset to treatment; the passing of time, represented in evolving observations of illness and health care experiences; and subtle references to historic time, which places the patient’s experience within broader societal trends or health care issues [22]. Complex, highly customized programming would be required to automate the extraction of time-related meaning from these texts.

Unsolicited online narratives, unlike interview data, contain large amounts of content that may be extraneous to the core interests of the research. Integrating deductive and inductive methods analysis [23] allowed us to efficiently extract relevant data while allowing for unexpected insights. In NVivo, the data were first categorized into a basic deductive framework [23] comprising the 2 key domains of care assessed in patient-reported experience measures: functional (clinical care and the practical delivery of services) and relational (relationships with clinicians and health care service providers) experiences of care. We then inductively sought emergent themes in the data categorized into each of these domains, following the conventions established by Braun et al [24].

Finally, we propose a narratological component in the qualitative analysis because personal experience is conveyed not only through story content but also through the choices made in storytelling [25,26]. Our narratological component examined the storytelling structure used by the authors. Typically, this structure followed Western narrative conventions: a prologue depicting life before the first symptom, the main story describing events leading up to diagnosis, and an epilogue expressing closure to the story [27]. We observed that epilogues served as spaces for authors to reflect on lessons learned and the broader implications of their experiences. Our epilogue-only analysis identified a substantial set of patient-reported views on the need for self-advocacy and changes to the views of clinicians on colorectal cancer among young people. These findings were published in a second paper arising from the study [7] and were used in a campaign by Bowel Cancer Australia to lower the screening age for bowel cancer from 50 to 45 years.

### Conclusions

Noninteractive online narratives can provide a singular, comprehensive perspective on patient journeys, capturing transitions across health care settings and services. As we found in our study of 273 noninteractive online narratives, this perspective offers researchers unique insights into systemic delays and gaps in continuity. In our study, we demonstrated the value of a framework tailored to address the specific challenges and limitations of noninteractive data throughout the research cycle. The 5 focal points of the framework offer a model for researchers navigating the complexities of these data.

In future work, utilizing this framework can ensure the integrity and value of exploratory studies that seek to identify emerging issues exposed in longitudinal patient accounts published online. The framework may provide collaborative stakeholders—researchers, health care providers, policy makers, and patient advocates—with a structured means of understanding how credible, patient-centered insights be drawn from these data. Additionally, the 5 focal points are adaptable to new digital environments for patient narratives and advanced analytical technologies. In these ways, the framework reinforces the potential for online patient journey narratives to inform health care research and affirms that patients’ stories can guide solutions to health care problems.

## Abbreviations

IMR: Internet Mediated Research

## Data Availability

The datasets used and/or analyzed during this study are available from the corresponding author on reasonable request.
